# Processing speed impairment in chronic inflammatory demyelinating polyneuropathy patients: a cross-sectional study

**DOI:** 10.1055/s-0042-1758557

**Published:** 2022-12-19

**Authors:** Iara Senem, Carina Telarolli Spedo, Rodrigo Melo Conde, Geraldo Cassio dos Reis, Antônio Carlos dos Santos, Amilton Antunes Barreira, (in memoriam), Wilson Marques Jr

**Affiliations:** 1 Universidade de São Paulo, Faculdade de Medicina de Ribeirão Preto, Departamento de Neurociências e Ciências do Comportamento, Ribeirão Preto SP, Brazil.; 2 Universidade Federal de São Carlos, Departamento de Psicologia, São Carlos SP, Brazil.; 3 Faculdade Anhanguera, Departamento de Fisioterapia, Ribeirão Preto SP, Brazil.

**Keywords:** Polyradiculoneuropathy, Chronic Inflammatory Demyelinating, Cognition, Executive Function, Polirradiculoneuropatia Desmielinizante Inflamatória Crônica, Cognição, Função Executiva

## Abstract

**Background** There is a lack of evidence of cognitive involvement in chronic inflammatory demyelinating polyneuropathy (CIDP) and, the reports about the involvement of the brain and central nervous system (CNS) are few and controversial. The Five Digit Test (FDT) evaluates processing speed (PS) and executive functions orally.

**Objective** To evaluate the performance on the FDT of CIDP patients with and without CNS (brain/cerebellum) alterations observed on brain Magnetic Resonance Imaging (MRI) scans.

**Methods** The Hospital Anxiety and Depression Scale (HADS, to assess neuropsychiatry symptoms), the Rasch-built Overall Disability Scale (R-ODS; to assess disability), and the FDT (to assess cognition) were applied to 14 CIDP patients and 24 age-matched healthy control subjects. The patients were submitted to routine brain MRI and, according to the results, they were divided into two groups: those with abnormalities on the MRI (CIDPabnl) and those with normal parameters on the MRI (CIDPnl). The FDT data of five CIDPnl patients and nine CIDPabnl subjects were analyzed. Comparisons between the groups were performed for each task of the FDT.

**Results** We found statistical differences for both groups of CIDP patients in terms of PS, for the patients spent more time performing the PS tasks than the controls. The PS measures were negatively associated with disability scores ( *reading* : r = −0.47; *p* = 0.003; *counting* : r = −0.53; *p* = 0.001).

**Conclusions** Our data suggested the presence of PS impairment in CIDP patients. Disability was associated with slow PS.

## Resumo

**Antecedentes** Faltam evidências de envolvimento cognitivo na polineuropatia inflamatória desmielinizante crônica (PIDC), e há poucos e controversos estudos que tratam do envolvimento cerebral e do sistema nervoso central (SNC). O Teste dos Cinco Dígitos (Five Digit Test, FDT, em inglês) avalia a velocidade de processamento (VP) e as funções executivas oralmente.

**Objetivo** Avaliar o desempenho no FDT de pacientes com PIDC com e sem alterações no SNC (cérebro/cerebelo) de acordo com o exame de imagem cerebral por ressonância magnética (RM).

**Métodos** Ao todo, 14 pacientes e 24 controles saudáveis pareados por idade responderam a Escala Hospitalar de Ansiedade e Depressão (que avalia sintomas neuropsiquiátricos), a Escala de Incapacidade Geral elaborada pelo método Rasch (que avalia a incapacidade) e o FDT (que avalia a cognição). Os pacientes foram submetidos a RM cerebral e, de acordo com os resultados, divididos em dois grupos: aqueles com anormalidades (PIDCabnl) e aqueles sem alterações (PIDCnl) na RM. Cinco pacientes PIDCnl e nove PIDCabnl tiveram os dados analisados. Comparações entre os grupos foram realizadas para cada parte do FDT.

**Resultados** Os dois grupos de pacientes foram estatisticamente mais lentos nas tarefas de VP comparados ao grupo controle. As medidas de VP foram negativamente associadas às pontuações de incapacidade ( *leitura* : r = −0,47; *p* = 0,003; *contagem* : r = −0,53; *p* = 0,001).

**Conclusões** Os dados indicaram a presença de prejuízo na VP em pacientes com PIDC. A incapacidade foi associada à lentidão na VP.

## INTRODUCTION

Chronic inflammatory demyelinating polyneuropathy (CIDP) is an autoimmune disease of the peripheral nerve system (PNS). 1 Its prevalence varies according to geographic region, ranging from 1 to 9 cases in every 100,000 habitants, and affecting males more than females. 2 3 Its presentation is heterogeneous, and effective biomarkers have not been found. 3 4 According to the recently-published criteria by the Joint Task Force of the European Federation of Neurological Societies and the Peripheral Nerve Society (EFNS/PNS), 5 the typical CIDP affects symmetrically distal and proximal muscles with sensory involvement of at least two limbs, whereas the CIDP variants include various subtypes with a distinct clinical and electrodiagnostic phenotype. 5 It is usually responsive to intravenous human immunoglobulin (IVIg), plasmapheresis, and immunosuppressants, indicating its autoimmune nature. 6 The prognosis depends on the extension of the nerve damage, and it seems unfavorable when there is concomitant involvement of the central nervous system (CNS). 7

Abnormalities in the CNS on magnetic resonance imaging (MRI) scans have been reported in patients with CIDP, 2 8 9 10 11 12 13 and some studies 9 14 suggest a possible association with multiple sclerosis (MS) due to the presence of white matter (WM) abnormalities, although no consensus has been reached. In the literature, the presence of CNS abnormalities on MRI is quite variable, ranging from 5% to 50% of the cases, 8 9 10 11 12 and the consequences of these abnormalities have not been properly addressed.

Processing speed (PS) is the mental speed to operate cognitive functions. 15 Studies 16 17 18 19 have assumed that PS is a mediating variable to perform cognitive functions, such as memory and intelligence competence. Additionally, some studies have indicated that PS measures decrease in some psychiatric and development disorders, 20 21 in human immunodeficiency virus (HIV) and hepatitis C virus infection, 22 in mild cognitive impairment (MCI), 23 and in MS, in which PS is usually severely compromised, and is associated with a decline in cognitive functions. 24 Regarding CIDP and cognition performance, we only found two studies addressing this subject. One of them 25 found PS deficits in CIDP, but there was no evaluation of CNS integrity. More recently, a study 26 evaluated cognition in a group of patients with chronic autoimmune demyelinating polyneuropathies (CADP) in general, and the authors detected a decrease in PS ability. Based on the literature, we hypothesized that the PS could be an essential domain to be investigated in CIDP patients according to the presence or absence of abnormalities on brain MRI scans.

The Five Digit Test (FDT) was developed by Sedó 27 as an instrument based on the Stroop paradigm, and it is used to evaluate the PS and some domains of executive functions. The FDT consists of reading and counting numbers and asterisks as fast as possible. 28 Slowness in the FDT indicates cognitive damage. 27 To the best of our knowledge, this instrument has not yet been used to evaluate cognition in CIDP. It has the advantage of not demanding hand movements, which are often impaired in CIDP patients. The present study aimed to evaluate the FDT performance of CIDP patients according to MRI results. Secondly, through a statistical analysis, we aimed to determine if patients without MRI abnormalities could present cognitive impairment measured by the FDT and compare them with patients with alterations on MRI and controls. The confirmation of any cognitive impairment in CIDP will increase knowledge and improve the health care and follow-up provided to these patients.

## METHODS

### Participants and procedures

The Research Ethics Committee of Faculdade de Medicina de Ribeirão Preto, Universidade de São Paulo, Brazil, approved the project (under protocol no. 10.333), and all participants signed an informed consent form before the experiment were started. The data were collected between July 2018 and April 2019 at the teaching hospital of the aforementioned university. A registered psychologist (I.S.) with a specialization in neuropsychology conducted the neuropsychological evaluation.

Healthy individuals from the community composed the control group (CG). The inclusion criteria were: preserved independence and functionality; no history of visual and auditory deficits; no history of any psychiatric or neurological disorders; not taking any medication that may impact cognition; and no history of alcoholism or drug abuse. We excluded one volunteer as she declared the use of medicines for migraine without a proper prescription.

The inclusion criteria for CIDP patients were: CIDP diagnosis according to the Joint Task Force of the EFNS/PNS; ^5^ absence of psychiatric diseases, except for anxiety and depression; no history of alcoholism or drug abuse; no intellectual disability; and age between 21 and 59 years. An expert neurologist (W.M. Jr) reviewed and confirmed all the diagnoses according to the new recently published criteria. 5

The recruitment of the CIDP patients began with an extensive search in the electronic database of our hospital. We initially found 240 patients registered as having CIDP. Only 31 of them were available and fulfilled the inclusion criteria, and 16 agreed to participate, and undergo an MRI. Only 14 finished the MRI evaluation, as 1 patient was excluded due to head injury, and another was unable to perform the tests. Only the patient group underwent the brain MRI. The MRI analysis was performed by a neuroradiologist (A.C.S), who wrote a report. The neuroradiologist had no information about the results of the neuropsychological evaluation.

### Materials

#### *Hospital anxiety and depression scale (HADS)*

The Hospital Anxiety and Depression Scale (HADS) is a self-report instrument used to evaluate symptoms of depression and anxiety. Previous studies 29 indicated the cutoffs for the Brazilian version. Due to the sample size and to compare the groups, we kept all participants, regardless of the cutoff point.

#### *Rasch-built overall disability scale (R-ODS)*

The version of the Rasch-built Overall Disability Scale (R-ODS) translated to Brazilian Portuguese was used to measure the degree of disability. The R-ODS is applied in cases of immune-mediated peripheral neuropathies, 30 and it is a self-report scale with 24 items about daily activities. The alternatives for the answers vary according to the degree of difficulty, and the maximum score is 48. The lower the score, the higher the degrre of disability.

#### *FDT*

The FDT evaluates the PS and executive function. 27 The adopted Brazilian Portuguese version 31 was used in the present research. The FDT scores are expressed in seconds. In the presence of marked sluggishness, the existence of neurocognitive difficulty is confirmed. 31 The FDT includes the following tasks: reading and counting (automatic processes), choosing and shifting (executive functioning), and inhibition and flexibility. 28 . Reading and counting are measures of mental speed (PS), while choosing and shifting are controlled, demanding high-order functions (executive function). The inhibition and flexibility parts are calculated after the subject finishes the test and are measured without the PS component using subtractions. 31

#### *Magnetic resonance imaging (MRI)*

The report from the routine brain MRI was used as a criterion to separate patients into two groups: those with (classified as CIDPabnl) and those without (classified as CIDPnl) CNS abnormalities (specifically in the brain and cerebellum). The 3T MRI scans included three-dimensional T1-weighted images, three-dimensional fluid attenuated inversion recovery (FLAIR-3D), axial T2-weighted images, diffusion tensor imaging (DTI) measurements, and susceptibility-weighted imaging (SWI).

### Statistical analysis

Statistical analyses were performed by an expert statistician (G.C.R) using the Statistical Package for the Social Sciences (SPSS, SPSS Inc., Chicago, IL, United States) software, version 13.0. The sociodemographic and clinical data were expressed as frequencies and percentages. Descriptive statistics were expressed as means, standard deviations and ranges. We used the chi-squared (χ ^2^ ) test and the Kruskal-Wallis nonparametric test to calculate the differences between the three groups. For the variables with a significant statistical difference among the groups, we applied the Dunn post hoc test to identify where these differences occurred. Then, we used the linear regression analysis method to adjust the variables that had a significant difference for years of schooling to verify whether the influence of groups remained in the cognitive variable, even after the adjustment.

We performed the Spearman rank correlation to test the association of FDT tasks with the other variables: age, years of schooling, disease duration, degree of disability (measured by the R-ODS), and symptoms of anxiety and depression (measured by HADS scores).

## RESULTS

Table 1 shows the characteristics of the three groups: CIDPabnl (n = 9), CIDPnl (n = 5), and the controls (n = 24). All patients fulfilled the new criteria for typical CIDP. 5 None of the patients had manifestations that could suggest nodo-paranodopathy.

**Table 1:** Characteristics of the participants of the present study

	Total sample	CIDPnl	CIDPabnl	CG	Group differences	Dunn post hoc
N	38	5	9	24	−	−
Male gender: n (%)	22 (57.9%)	4 (80.0%)	6 (66.7%)	12 (50.0%)	χ ^2^ ; *p* = 0.45	−
Age in years	41.66 (12.04) [24-59]	42.0 (13.95) [24-56]	46.22 (12.18) [24-58]	39.88 (11.67) [24-59]	χ ^2^ = 1.53; *p* = 0.46	−
Years of schooling	14.39 (4.42) [3-24]	11.0 (4.90) [3-15]	11.11 (4.04) [4-15]	16.33 (3.32) [11-24]	χ ^2^ = 11.36; *p* = 0.003*	CG > CIDPabnl: 0.009*
Disease duration (years)	−	9.20 (5.21) [2-16]	11.67 (10.61) [2-38]	−	χ ^2^ = 0.02; *p* = 0.89	−
HADS A	6.82 (3.41) [1-14]	7.0 (3.67) [4-11]	8.67 (3.61) [5-14]	6.08 (3.16) [1-14]	χ ^2^ = 3.51; *p* = 0.17	−
HADS D	4.92 (3.42) [0-13]	5.0 (3.46) [2-10]	5.11 (4.04) [0-12]	4.83 (3.33) [1-13]	χ ^2^ = 0.01; *p* = 0.99	−
R-ODS	47.74 (4.84) [29-50]	48.0 (2.55) [44-50]	42.0 (7.38) [29-50]	49.83 (0.38) [49-50]	χ ^2^ = 20.31; *p* < 0.001*	CG > CIDPabnl < 0.001*; CG > CIDPnl 0.05*

**Abbreviations:** χ ^2^ , Chi-squared test; A, anxiety domain of the HADS; CG, control group; CIDP, chronic inflammatory demyelinating polyneuropathy; CIDPabnl, patients with abnormalities on brain MRI; CIDPnl, patients with normal parameters on brain MRI; D, depression domain of the HADS; HADS, Hospital Anxiety and Depression Scale; R-ODS, Rasch-built Overall Disability Scale.

Notes: Results are presented as mean (standard deviation [SD]) [range], except for gender. * *p* < 0.05.

### Clinical characteristics of the CIDPnl

The CIDPnl group was composed of 4 men and 1 woman aged between 24 and 56 (mean = 42; standard deviation [SD] = 13.95) years. All patients were right-handed. One patient who was a smoker presented trigeminal nerve thickening on the right side on the brain MRI. One patient had systemic arterial hypertension (SAH), and another had monoclonal gammopathy of undetermined significance (MGUS) as a comorbidity. At the time of the evaluation (the previous three months), four patients were not receiving treatment for CIDP. The patients interrupted therapy on their own. One patient was receiving IVIg. Table 2 shows the clinical data for the CIDPnl.

**Table 2:** Clinical characteristics of CIDPnl patients

n = 5	Cranial nerves involved	Other conditions	Current treatment*
Patient 1	−	−	−
Patient 2	Trigeminal nerve thickening to right	−	−
Patient 3	−	−	−
Patient 4	−	Systemic arterial hypertension	−
Patient 5	−	Monoclonal gammopathy of undetermined significance	Intravenous immunoglobulin therapy

Note: *The previous three months. Untreated patients had not been receiving medication in the past three months. These patients interrupted treatment on their own.

### Clinical characteristics of the CIDPabnl

**Figure 1.**
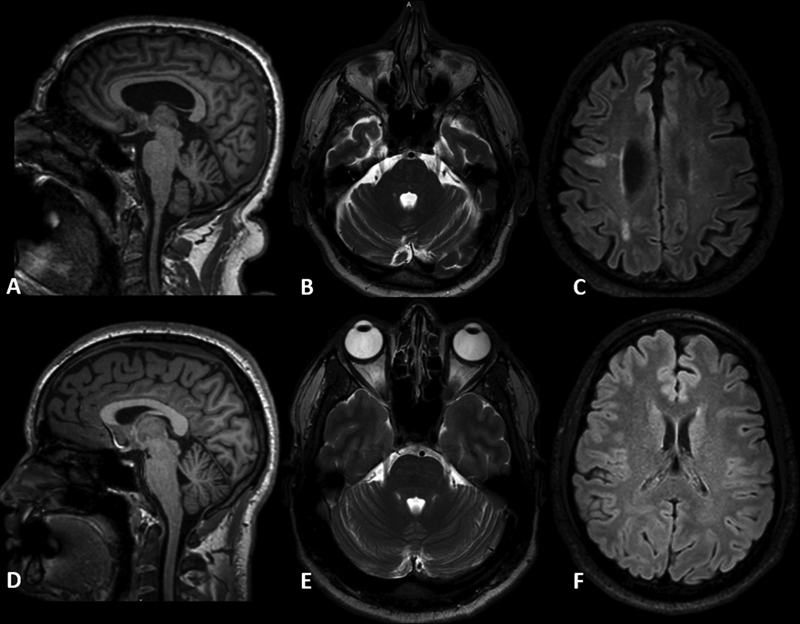
Sagittal and axial brain MRI scans of two CIDP patients. ( **A-C** ) Structural damage: brain and cerebellar atrophy in a patient classified as CIDPabnl (patient 6). ( **D-F** ) Normal MRI of one CIDP patient with normal parameters in MRI (patient 1).

The CIDPabnl group was composed of 9 individuals aged between 24 and 58 (mean = 46.22; SD = 12.18) years. One patient was left-handed, and none of them reported smoking. One patient had SAH, and another one had MGUS. None of the patients met the criteria for MS. According to the MRI, two patients had trigeminal nerve thickening (patients 3 and 5; Table 3 ). Surprisingly, eight patients had some degree of cerebellar atrophy. Besides, microangiopathy was observed in 2 patients with concomitant cerebellar atrophy (patients 6 and 8). Patient 6 also presented brain atrophy. Occipital demyelination was described in one patient. Patient 2, who was 24 years old and severely impaired (difficulty walking and weak hands), presented a reduction in corpus callosum thickness. Five patients were treated with IVIg, and 2 patients were on corticosteroid therapy (patients 3 and 5). Two patients were taking gabapentin or carbamazepine to relieve pain (patients 4 and 7). Table 3 displays the clinical characteristics of the CIDPabnl. Figure 1 shows the brain MRI of two patients, one classified as CIDPnl and another, as CIDPabnl (patients 1 and 6 respectively).

**Table 3:** Characterization of the MRI report of the CIDPabnl group

n = 9	MRI report/CNS involvement	Cranial nerves involved	Other conditions	Current treatment*
Patient 1	Cerebellar atrophy	−	Systemic arterial hypertension	Intravenous immunoglobulin
Patient 2	Mild cerebellar atrophy, reduction in corpus callosum thickness	−	−	Intravenous immunoglobulin
Patient 3	Cerebellar atrophy	Trigeminal nerve	Monoclonal gammopathy of undetermined significance	Methylprednisolone
Patient 4	periarteriolar demyelinatin features	−	−	Gabapentin
Patient 5	occipital demyelination	Trigeminal nerve	−	Methylprednisolone
Patient 6	Cerebellar atrophy, brain atrophy, microangiopathy	−	−	Intravenous immunoglobulin
Patient 7	Cerebellar atrophy	−	−	Carbamazepine
Patient 8	Cerebral parenchyma, microangiopathy	−	−	Intravenous immunoglobulin
Patient 9	Cerebellar atrophy	−	−	Intravenous immunoglobulin

Note: *The last three months; Only the principal treatment is described.

### Differences between groups

There were no differences among the groups regarding gender distribution, age and HADS scores ( Table 1 ). The sample was statistically different in terms of years of schooling, and the value for the CG was significantly higher than that of CIDPabnl ( *p* = 0.009). The disability measure (R-ODS) presented significant difference among the groups, with the controls performing better than both the CIDPabnl ( *p* < 0.001) and CIDPnl ( *p* = 0.05).

Table 4 shows the FDT data. The case groups had a statistically inferior performance than the controls regarding automatic process measures (reading and counting). The controls were statistically faster in the FDT choosing task than the case groups. In the FDT shifting, we found differences between the CIDPabnl and the CG ( *p* = 0.03). In the flexibility and inhibition measures, there were no differences. After controlling for years of schooling, the differences remaining for the FDT were: reading for the CIDPnl ( *p* = 0.03), counting for the CIDPabnl ( *p* = 0.001), and choosing for the CIDPnl ( *p* = 0.004).

**Table 4:** Data from the Five Digit Test

FDT tasks	CIDPnl (n = 5)	CIDPabnl (n = 9)	CG (n = 24)	Non-parametric test comparing groups ^a^		Adjustment for schooling by the linear regression model ^b^		
				Group differences	Dunn post hoc		Beta	*p*
**Processing speed – automatic process measures**								
Reading	31.8 (7.5) [22-39]	29.22 (6.06) [24-44]	22.08 (4.54) [13-28]	χ ^2^ = 13.21; *p* = 0.001*	CIDPabnl > CG: 0.006*; CIDPnl > CG: 0.05	CIDPabnl	0.23	0.12
						CIDPnl	0.31	0.03*
Counting	30.4 (2.97) [26-33]	33.89 (7.02) [27-46]	24.0 (3.88) [17-32]	χ ^2^ = 18.41; *p* < 0.001*	CIDPabnl > CG: < 0.001*; CIDPnl > CG: 0.02*	CIDPabnl	0.45	0.001*
						CIDPnl	0.16	0.18
**Executive functions – controlled process measures**								
Choosing	61.8 (28.92) [40-110]	49.67 (12.24) [32-69]	37.83 (7.73) [24-52]	χ ^2^ = 10.69; *p* = 0.005*	CIDPabnl > CG: 0.03*; CIDPnl > CG: 0.05	CIDPabnl	0.02	0.91
						CIDPnl	0.27	0.04*
Shifting	76.4 (42.58) [45-148]	61.67 (17.76) [41-99]	49.75 (11.76) [29-77]	χ ^2^ = 6.21; *p* = 0.05	CIDPabnl > CG: 0.03*	CIDPabnl	−0.53	0.73
						CIDPnl	0.19	0.2
Inhibition	30 (23.91) [15-72]	20.44 (9.19) [8-38]	15.79 (7.18) [2-29]	χ ^2^ = 3.55; *p* = 0.16	−	−	−	−
Flexibility	44.6 (37.6) [19-110]	32.44 (15.52) [17-70]	27.67 (11.45) [14-52]	χ ^2^ = 1.81; *p* = 0.40	−	−	−	−
Shifting errors	2.2 (3.19) [0-7]	2.33 (2.64) [0-8]	0.37 (0.71) [0-2]	χ ^2^ = 6.7; *p* = 0.03*	CIDPabnl > CG: 0.05	CIDPabnl	0.21	0.21
						CIDPnl	0.14	0.37

**Abbreviations:** χ ^2^ , Chi-squared test; CG, control group; CIDP, chronic inflammatory demyelinating polyneuropathy; CIDPabnl, CIDP with abnormal brain MRI; CIDPnl, CIDP with normal brain MRI; FDT, Five Digit Test.

Notes: Results are presented as mean (standard deviation [SD]) [range]. ^a^ Kruskal-Wallis and Dunn post hoc tests. ^b^ Only performed when there was a significant difference. The errors in reading, counting, and choosing were not presented. There were no statistically significant dofferences regarding errors in reading, counting and choosing. * *p* < 0.05.

### Correlation analysis

Table 5 displays all correlation analyses performed. We found significant and positive correlations between age and FDT counting (r = 0.36; *p* = 0.03), age and FDT shifting (r = 0.33; *p* = 0.04), and age and FDT flexibility (r = 0.34; *p* = 0.04). Years of schooling presented negative correlations with all FDT domains. There was no significant correlation between FDT and disease duration. The R-ODS score was negatively correlated with FDT reading (r = −0.47; *p* = 0.003), FDT counting (r = −0.53; *p* = 0.001) and FDT choosing (r = −0.41; *p* = 0.01). Regarding anxiety and depression symptoms, we found only one significant correlation with anxiety scores (r = 0.37; *p* = 0.02).

**Table 5:** Correlations between FDT domains and measures of disability, anxiety symptoms, depressions symptoms, and years of schooling

n = 38	Schooling	R-ODS	HADS A	HADS D
FDT reading	r = −0,50; *p* = 0.001*	r = −0.47; *p* = 0.003*	r = 0.06; *p* = 0.72	r = −0.07; *p* = 0.69
FDT counting	r = −0.68; *p* < 0.001*	r = −0.53; *p* = 0.001*	r = 0.37; *p* = 0.02*	r = 0.16; *p* = 0.32
FDT choosing	r = −0.65; *p* < 0.001*	r = −0.41; *p* = 0.01*	r = 0.11; *p* = 0.51	r = −0.02; *p* = 0.89
FDT shifting	r = −0.60; *p* < 0.001*	r = −0.19; *p* = 0.26	r = 0.07; *p* = 0.69	r = 0.08; *p* = 0.62
FDT inhibition	r = −0.50; *p* = 0.002*	r = −0.15; *p* = 0.37	r = 0.10; *p* = 0.56	r = 0.02; *p* = 0.90
FDT flexibility	r = −0.38; *p* = 0.02*	r = 0.02*; *p* = 0.89	r = 0.01*; *p* = 0.93	r = 0.10; *p* = 0.57

Abbreviations: A, anxiety domain of the HADS; D, depression domain of the HADS; FDT, Five Digit Test; HADS, Hospital Anxiety and Depression Scale; R-ODS, Rasch-built Overall Disability Scale.

Note: *Significant rank-order correlation at *p* < 0.05.

## DISCUSSION

One crucial measure evaluated by the FDT is the PS, which may be slowed in neurological conditions. 17 18 19 As there is no demand for hand function to complete this test, it is particularly useful in CIDP. The purpose of the present study was to evaluate cognitive function in CIDP measuring the PS and executive function through the FDT in patients with normal and abnormal brain images. The main findings of the present study were: both case groups presented lower PS; the abnormalities found on MRI did not explain the observed slowdown in the PS; and there was no difference between patients and controls in the flexibility and inhibition domains, which are measures derived after exclusion of PS interference.

Deficits in PS in CIDP patients have already been reported, 25 and they are attributed to CNS damage. The present study however, demonstrated that PS underperformance was also present in patients with normal brain MRIs. Together, these findings support the presence of slowness in cognitive processing in CIDP patients, regardless of visible abnormalities on brain MRI scans. Moreover, a recent investigation 27 linked the decline in PS to a dysfunction in the blood-brain barrier (BBB) in certain CADP patients. Similarly, inflammatory conditions have been described as impacting PS and cognition. 32 A similar mechanism may exist in CIDP. Inflammatory mechanisms could be present in our CIDPnl patients, impacting PS without evidence of structural changes on routine MRI. Future research could investigate inflammatory markers associated with cognitive functions in CIDP.

During the FDT tasks, the subject looks at the stimuli and emits a verbal response, which was slow in the patient groups. It is possible that changes in the peripheral input may play a role. Studies 33 with visual evoked potential (VEP) indicated abnormalities in some CIDP patients. Another VEP study 34 recently revealed the possibility of central sensory involvement correlated with the degree of peripheral nerve impairment in CIDP. This could indicate that the damage in the periphery impacts PS, increasing the time to accomplish the task. To confirm it, the VEP should be performed in future studies. Thus, one hypothesis is that the PNS damage decreases the afferent impulses, reducing the necessary drive to trigger a fast voluntary motor response.

The FDT choosing task is a measure of attention, a component of executive functions. After controlling for years of schooling, only the CIDPnl group presented lower values. This finding confirms a preliminary report 35 in which the authors examined seven CIDP patients with normal MRI scans and demonstrated deficits in selective and divided attention. In the present study, the first statistical analysis showed differences in FDT shifting for the CIDPabnl, but no difference was found when controlled for years of schooling. It was an unexpected result, since almost all CIDPabnl patients presented cerebellar atrophy (except patient number 8). The extension of the cerebellar atrophy may not be enough to reflect on the executive function domains in this sample. This could be investigated in future research, since the cerebellum has been implicated in executive functions. 36 Also, two out of nine CIDPabnl patients were taking corticosteroids. It is recognized that corticosteroids can impact cognitive abilities, especially executive function, 37 but this was not reflected in our sample. Our data on the executive functions measured by the FDT were heterogeneous. They may reflect the educational characteristics of the sample or point to heterogeneity in cognitive domains.

The inhibition and flexibility measures, which were calculated without PS interference, were diminished in both groups of patients. This could reinforce the PS as a central deficit in CIDP, indicating the need for follow-up and the possibility of subclinical damage. Also, the disability was negatively correlated with all FDT measures impacted after the correction for years of schooling (reading, counting, and choosing). This suggests that the PS performs a critical role in these patients. Future research could associate the FDT times with VEPs and the degree of peripheral nerve impairment.

One study 38 reported depression in CIDP patients; in the present study, there was no significant difference between patients and controls regarding to the anxiety and depression domains of the HADS. Note that, in the present study, some participants had exceeded the cut-off point for the HADS, but we kept them in the statistical analysis because there was no difference among the groups. Previous studies had reported that the mood could negatively affect PS measures, 39 but there was only one correlation between the anxiety domain of the HADS and FDT counting in our sample. For future research, it is crucial to consider these variables.

This present study is the first to analyze FDT performance in CIDP patients and to compare the possible alterations in the brain/cerebellum according to routine MRIs. However, our research has some limitations: the first is the difference in the level of schooling among the groups. We had difficulty getting a paired control group. When we statistically controlled this variable, the results were heterogeneous for the executive function domain. Secondly, the sample size is small, and we cannot perform statistical analyses to evaluate the association between therapy type and FDT performance. Third, we kept all participants regardless of the cut-offs for the HADS due to our sample size. We consider that data DTI and VEP data and their correlation with the PS measure could be used in future research. Additionally, studies correlating BBB integrity and PS could be carried out with a longitudinal and multicenter design. We emphasize that neuromuscular, autoimmune, and demyelinating disorders can present neuropsychological aspects that should be considered by health professionals.

In conclusion, the present study provides preliminary evidence for the slowness in PS tasks of the FDT in CIDP patients. Since some CIDP patients have limitations regarding hand movements, the FDT seems be useful to evaluate PS and executive function in this population. Our results suggest that PS is a relevant domain to be evaluated in these patients. Longitudinal studies are necessary to evaluate PS as subclinical damage in this population.
